# Telomere Shortening in Peripheral Leukocytes Is Associated With Poor Survival in Cancer Patients Treated With Immune Checkpoint Inhibitor Therapy

**DOI:** 10.3389/fonc.2021.729207

**Published:** 2021-08-19

**Authors:** Benjamin Rolles, Joao Gorgulho, Mareike Tometten, Christoph Roderburg, Margherita Vieri, Anne Abels, Mihael Vucur, Felix Heymann, Frank Tacke, Tim H. Brümmendorf, Tom Luedde, Fabian Beier, Sven H. Loosen

**Affiliations:** ^1^Department of Hematology, Oncology, Hemostaseology and Stem Cell Transplantation, Medical Faculty, RWTH Aachen University, Aachen, Germany; ^2^Center for Integrated Oncology Aachen Bonn Cologne Duesseldorf (CIO ABCD) , Aachen, Germany; ^3^Department of Oncology, Haematology and Bone Marrow Transplantation with Section Pneumology, University Hospital Hamburg-Eppendorf, Hamburg, Germany; ^4^Clinic for Gastroenterology, Hepatology and Infectious Diseases, University Hospital Düsseldorf, Medical Faculty of Heinrich Heine University Düsseldorf, Düsseldorf, Germany; ^5^Department of Hepatology and Gastroenterology, Charité Universitätsmedizin Berlin, Berlin, Germany

**Keywords:** PD-L1, PD-1, CTLA-4, telomere length, tumor immunity

## Abstract

**Background:**

Immune checkpoint inhibitor (ICI) therapy represents a new standard of care for an increasing number of malignancies. Nevertheless, response rates and outcome of ICI treatment vary between individuals and the identification of predictive markers or hints towards immune cell exhaustion during therapy has remained a major challenge. Leukocyte telomere length is an established predictive biomarker of replicative aging and cellular proliferative potential in various hematological diseases. However, its relevance in the context of ICI therapy has not been investigated to date. Here, we analyze the age-adapted delta telomere length (ΔTL) of peripheral leukocytes as a potential predictive and prognostic marker in patients undergoing ICI therapy.

**Methods:**

Age-adapted delta telomere length (ΔTL) of 84 patients treated with ICIs for solid malignancies was measured *via* quantitative real-time PCR. ΔTL was correlated with outcome and clinical data.

**Results:**

ΔTL was not significantly altered between patients with different tumor entities or tumor stages and did not predict tumor response to ICI therapy. However, ΔTLs at initiation of treatment were a prognostic marker for overall survival (OS). When using a calculated ideal cut-off value, the median OS in patients with shorter ΔTL was 5.7 months compared to 18.0 months in patients showing longer ΔTL. The prognostic role of age-adapted ΔTL was further confirmed by uni- and multivariate Cox-regression analyses.

**Conclusion:**

In the present study, we demonstrate that shorter telomere lengths in peripheral blood leukocytes are associated with a significantly impaired outcome in patients receiving ICI therapy across different malignancies. We explain our findings by hypothesizing an older replicative age in peripheral leukocytes of patients with an impaired overall survival, reflected by a premature TL shortening. Whether this association is ICI-specific remains unknown. Further follow-up studies are needed to provide insights about the exact mechanism of how shortened telomeres eventually affect OS and could help guiding therapeutic decisions in future.

## Introduction

Immune checkpoint inhibitors (ICI) have a wide range of medical applications in various malignancies including lung cancer (1), melanoma (2), renal cell carcinoma (3) and squamous-cell carcinoma of the head and neck (4). Among a large number of antibodies, nivolumab, pembrolizumab, and ipilimumab for combination therapy, represent the most frequently used checkpoint inhibitors. Local tissue cells, activated immune cells, as well as malignant cells are able to express programmed cell death ligand 1 (PD-L1) in order to influence tumor defense (5). Nivolumab and Pembrolizumab are antibodies that inhibit the programmed cell death protein 1 (PD-1) signaling, decrease PD-L1- and PD-L2-mediated immune silencing and ultimately lead to an increased anti-tumoral T cell activity, mainly mediated by cytotoxic T lymphocytes (6). Although ICIs have shown a high clinical efficacy in several studies, not all patients benefit from this new type of immunotherapy (7). The search for biomarkers that can help predict tolerance and response (including its long-time sustainability) to this promising type of therapy is ongoing. Previously, it has been shown that increased intratumoral expression of PD-L1 predicts a better treatment response to ICIs (8). Further studies indicate that tumors with microsatellite instability (MSI) seem to be characterized by superior response to immunotherapy using ICIs (9). Other factors such as the tumoral immune cell infiltration, tumor mutational burden, and some tumor specific mutations [e.g. BRAF-mutant non-small cell lung cancer (NSCLC) (10)], have also been associated with favorable treatment response (11). Nevertheless, predictive biomarkers of response per se as well as its long-term benefits have not yet been identified (11).

Telomeres are repetitive DNA sequences located at the ends of the chromosomes. Intact telomeres are essential for genomic integrity and the maintenance of the cellular proliferation potential (12). During life, telomeres shorten with each cell division and somatic cells become senescent or undergo apoptosis once reaching a critically short TL (13). TL represents an established biomarker for the replicative history in specific tissues, turnover of hematopoietic stem cell populations (14–16) as well as leukocyte subsets (14, 17) and the biological aging process of an individual organism in general (18). For various hematological diseases (13), TL has been shown to predict treatment response, such as in chronic myeloid leukemia (19, 20), or has been demonstrated to correlate with the response to immunosuppressive therapy in aplastic anemia (13, 21). However, it currently remains unknown whether the individual TL plays a role in treatment response and outcome in patients undergoing ICI therapy. Thus, the primary aim of our study was to investigate whether the TLs of peripheral blood cells in subjects undergoing ICI therapy is a possible biomarker for therapy response and overall survival (OS).

## Materials and Methods

### Study Design and Patient Characteristics

84 patients receiving treatment with ICIs for solid malignancies at the interdisciplinary cancer outpatient clinic at the University Hospital Aachen of the RWTH Aachen University were included in this study between 2018 and 2020. The patient cohort was previously described to study novel biomarkers concerning ICI therapy (22, 23). The study protocol was approved by the ethics committee of the University Hospital RWTH Aachen, Germany (EK 206/09) and was conducted in accordance with the ethical standards laid down in the Declaration of Helsinki. Written informed consent was obtained from all patients. Detailed patient characteristics are shown in **Table 1**. For all patients we documented age, body-mass-index (BMI), underlying disease, disease stage, OS, objective response rates (ORR), adverse side effects, cycles and dosage of therapy as well as standard blood counts and serum markers.

**Table 1:** Patient characteristics.

Parameter	Study cohort
Cancer patients, no.	84
Gender [%]:	
male	66.7
female	33.3
Age, years [median and range]	68 [34 - 87]
BMI, kg/m^2^ [median and range]	24.2 [15.9 - 42.3]
Tumor localization [%]:	
NSCLC	40.5
Malignant melanoma	11.9
Urothelial cancer	11.9
GI cancer	15.5
Head and neck cancer	10.7
Others	9.5
Staging [%]:	
UICC III	7.3
UICC IV	92.7
ICI regimen [%]:	
Nivolumab	57.2
Pembrolizumab	28.6
Nivolumab + Ipilimumab	7.1
Others (e.g. Avelumab, Durvalumab)	7.1
Previous systemic therapy before ICI [%]:	
Yes	70.2
No	29.8
ECOG PS [%]:	
ECOG 0	4.8
ECOG 1	54.8
ECOG 2	36.9
ECOG 3	3.6
Smoking status [%]:	
Never	8.3
Previous	40.5
Present	20.2
Unknown	31.0
Disease control at 3 months [%]:	
Yes	42.9
No	57.1
Disease control at 6 months [%]:	
Yes	33.3
No	66.7
Disease control at 12 months [%]:	
Yes	26.5
No	73.5
Deceased during follow-up [%]:	
Yes	71.4
No	28.6
Side effects to ICI? [%]:	
Yes	39.3
No	60.7

No, number; BMI, body mass index; NSCLC, non-small cell lung cancer; GI, gastrointestinal; UICC, Union for International Cancer Control; ICI, immune checkpoint inhibitor; ECOG PS, Eastern Cooperative Oncology Group performance status.

### Blood Sample Acquisition

Blood samples were drawn before ICI treatment initialization or after the first administration of ICI. For the isolation of peripheral blood mononuclear cells (PBMCs), we drew blood using the BD Vacutainer^®^ CPT™ System (Cat No.:362782, BD Bioscience, USA), and followed manufacturer’s instructions for PBMC isolation (24). PBMCs were cryopreserved with 10% DMSO at −80°C until use.

### Assessment of Tumor Response and Overall Survival

Tumor response was evaluated by CT or MRI scan at three, six and 12 months after treatment initialization. The different tumor responses, complete response (CR), partial response (PR), stable disease (SD) or progressive disease (PD) were based on the evaluation of an experienced radiologist. OS was calculated from the first day of immunotherapy to death. CR, PR, and SD were subsumed as “disease control” (DC) whereas PD was classified as “no disease control” (nDC).

### Assessment of Immune-Related Adverse Events

Therapy tolerance of all patients was reviewed at regular intervals by an experienced oncologist. Immune-related adverse events (IRAEs) were separately documented and treated based on the recommendations of the American Society of Clinical Oncology (ASCO) (25). The severity was evaluated using the Common Terminology for Adverse Events (CTCAE) version 5.0.

### Telomere Length Measurement

DNA from peripheral blood cells was extracted and 1.4ng of genomic DNA was used for TL analysis. Measurement of TL was performed using the Absolute Human Telomere Length Quantification qPCR Assay Kit (ScienCell, Carlsbad, CA, USA) and FastStart Essential DNA Green Master (Roche, Basel, Switzerland). Quantitative real-time PCR (qPCR) was done with an ABI7500fast real-time PCR system (Applied Biosystems, Foster city, CA, USA) according to the Assay Kit protocol and described previously (26). TL was measured as T/S ratio. Peripheral blood samples of 104 healthy blood donors of various ages were used as control group. The healthy comparison population had a mean age of 43 years. The age range of subjects was from 18 years to 83 years. TL analysis was carried out single-blinded. Linear regression analysis was used to calculate the physiological telomere shortening during aging. For age-adaptation, patients’ measured TL was related to the respective calculated TL of the control population at the same age as described previously (20, 27). The calculated relative TL differences result in the “age-adapted delta telomere length” (ΔTL). Negative values correspond to a shortening of telomeres compared to the healthy reference population.

### Statistical Analysis

Shapiro-Wilk-Test was used to test for normal distribution. Non-parametric data were compared by Mann-Whitney-U-Test and the Kruskal-Wallis-Test. Box plot graphics display the median, quartiles and ranges. Kaplan-Meier curves show the impact of different ΔTL cut-off values on OS. The Log-rank test was used to test for statistical differences between subgroups in Kaplan-Meier curve analyses. To identify the optimal ΔTL cut-off for OS, we used Cox proportional hazard models to the dichotomized survival status as well as the survival and defined the optimal cut-off as the point with the most significant split in log-rank test. The prognostic value of variables was further tested by uni- and multivariate Cox regression analyses. Parameters with a p-value of <0.250 in univariate testing were included into multivariate testing. The hazard ratio (HR) and the 95% confidence interval are displayed. All statistical analyses were performed with SPSS 23 (SPSS, Chicago, IL, USA) and RStudio 1.2.5033 (RStudio Inc., Boston, MA, USA) (28). P-value of < 0.05 was considered as statistically significant (*p < 0.05; **p < 0.01; ***p < 0.001).

## Results

### Evaluation of TL in Relation to Patient Characteristics

**Figure 1:**
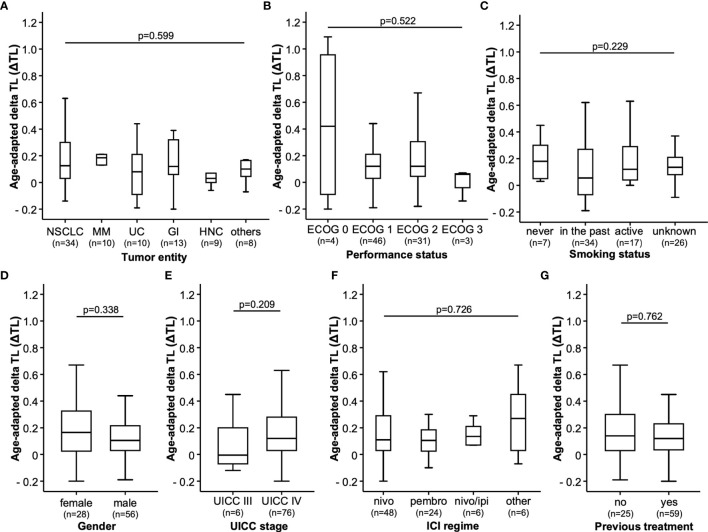
Study population. The age-adapted delta telomere length (ΔTL) is shown in patients with different **(A)** tumor entities [non-small cell lung cancer (NSCLC), malignant melanoma (MM), urothelial cancer (UC), gastrointestinal cancer (GI) head and neck cancer (HNC) and other tumor types (other)], **(B)** ECOG performance status, **(C)** smoking status, **(D)** gender of the patient, **(E)** advanced tumor stages classified by the UICC, **(F)** type of immunotherapeutics (nivolumab (nivo), pembrolizumab (pembro), a combination of nivolumab and ipilimumab (nivo/ipi) or other immunotherapeutics (other) and **(G)** if patients received pre-treatments (yes) or not (no).

Our cohort of 84 patients was analyzed for age-adapted ΔTL. Clinical characteristics of the study cohort are summarized in **Table 1**. We found no differences in ΔTL between patients with NSCLC, malignant melanoma (MM), urothelial carcinoma (UC), gastrointestinal cancers (GI), head and neck cancers (HNC) or other entities. There was a (non-significant) trend that ΔTL tended to be shorter in patients with HNC than in patients with MM (**Figure 1A**). Although overall median ΔTL was shortest in patients with ECOG 3 performance status, there was no significant difference between the different performance status (**Figure 1B**). In our cohort, smoking status had no impact on ΔTL, not even in actively smoking patients (**Figure 1C**). Furthermore, gender also had no impact on ΔTL (**Figure 1D**). All patients had an advanced tumor stage, with 6 patients in UICC stage III and 76 patients in UICC stage IV. Within that range, the UICC stage had no impact on ΔTL (**Figure 1E**). We also did not observe a significant difference in ΔTL in patients treated with nivolumab, pembrolizumab, the combination of nivolumab and ipilimumab or other ICIs (**Figure 1F**). Interestingly, whether patients had received other systemic therapies before ICI initialization had also no effect on patients’ ΔTL (**Figure 1G**).

### Evaluation of Treatment Response to ICI Therapy

**Figure 2:**
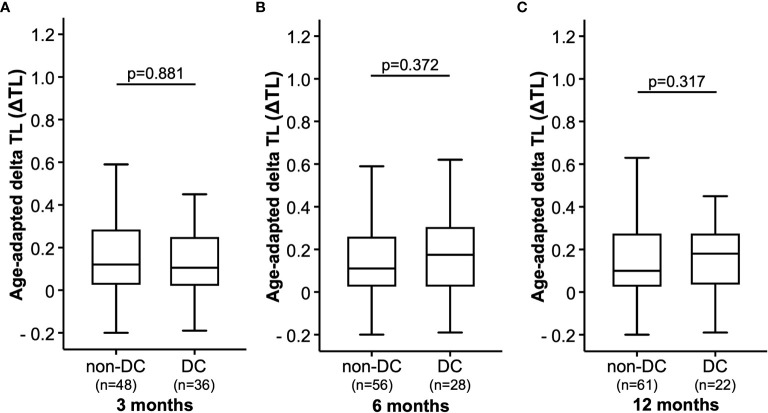
Therapy response. The age-adapted delta telomere length (ΔTL) is shown in patients with disease control (DC) or without disease control (non-DC) after **(A)** 3 months, **(B)** 6 months and **(C)** 9 months.

We next analyzed whether the patients’ ΔTL at baseline (ICI initiation) had an impact on treatment response to ICI therapy after three, six or 12 months. When looking at treatment response at three months, we observed comparable ΔTL values between patients who showed a controlled disease (DC, including patients with CR, PR, and SD) and patients with progressive disease (non-DC, **Figure 2A**). In line, the ΔTL value was unaltered between patients who did or did not show DC at six and 12 months, respectively (**Figures 2B, C**). Similar results were obtained, when applying a different categorization of tumor responders (responders: CR and PR *vs*. non-responders: SD and PD).

### A Shortened ΔTL Is Associated With an Impaired Overall Survival

**Figure 3:**
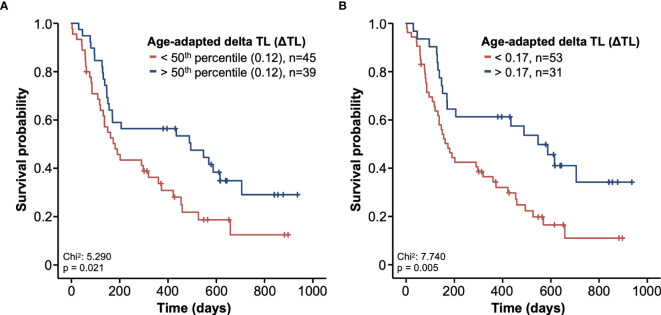
Kaplan-Meier curves of survival. The survival probability is shown in relation to the course of time. Patients were divided into two groups based on the ΔTL. **(A)** In the first graphic, the group was split based on the 50^th^ percentile. **(B)** In the second graphic, a relative telomere length of 0.17 was used as a cut-off. Patients with longer telomeres are shown in blue and patients with shorter telomeres are shown in red.

Based on previous findings of TL as a biomarker for treatment response (19, 20, 29), we next analyzed whether the individual ΔTL had an impact on the patients’ OS. Using the median ΔTL of all patients as a cut-off value (0.12), we observed a significantly impaired OS in patients with a ΔTL shorter than the cut-off value (**Figure 3A**). Subsequently, we established an ideal prognostic cut-off value of 0.17 *via* determination of the most significant split in the log rank test between patients with a good or poor outcome. Using this cut-off value, Kaplan-Meier curve analysis revealed a highly significantly impaired OS in patients with a ΔTL value below the ideal cut-off value (**Figure 3B**). The median OS in these patients was only 5.7 months compared to 18.0 months in patients with a median ΔTL above the ideal cut-off value. In a next step, we performed uni- and multivariate Cox-regression analyses to further substantiate the prognostic relevance of ΔTL in terms of OS. In univariate analysis, ΔTL turned out as a significant prognostic marker for OS (HR: 0.295, 95%CI: 0.092-0.950, p=0.041). When including other parameters of prognostic relevance in univariate analysis into multivariate Cox-regression analysis (BMI, ECOG PS, bilirubin and creatinine), the prognostic role of ΔTL was found to be independent of these confounders (HR: 0.280, 95%CI: 0.081-0.968, p=0.044, **Table 2**). While adaptation of TLs for age is a common practice, differences in OS remained significant even when based on non-age-adapted TLs (HR: 0.308; 95%CI: 0.099-0.958; p = 0.042).

**Table 2:** Uni- and multivariate Cox-regression analysis for the prediction of overall survival.

Parameter	Univariate Cox-regression		Multivariate Cox-regression	
	p-value	Hazard-Ratio (95% CI)	p-value	Hazard-Ratio (95% CI)
ΔTL	0.041	0.295 (0.092-0.950)	0.044	0.280 (0.081-0.968)
Age	0.806	0.997 (0.971-1.023)		
Sex	0.588	0.863 (0.506-1.470)		
BMI	0.007	0.928 (0.879-0.980)	0.022	0.936 (0.885-0.990)
UICC tumor stage	0.407	1.639 (0.510-5.267)		
ECOG PS	0.182	1.330 (0.875-2.020)	0.091	1.488 (0.939-2.359)
Leukocyte count	0.354	1.027 (0.970-1.088)		
Bilirubin	0.127	1.519 (0.888-2.599)	0.041	1.711 (1.023-2.862)
Creatinine	0.242	0.755 (0.472-1.08)	0.691	0.909 (0.567-1.457)

BMI, Body-Mass-Index; UICC, Union for International Cancer Control; ECOG PS, Eastern Cooperative Oncology Group performance status.

### IRAEs During ICI Therapy

**Figure 4:**
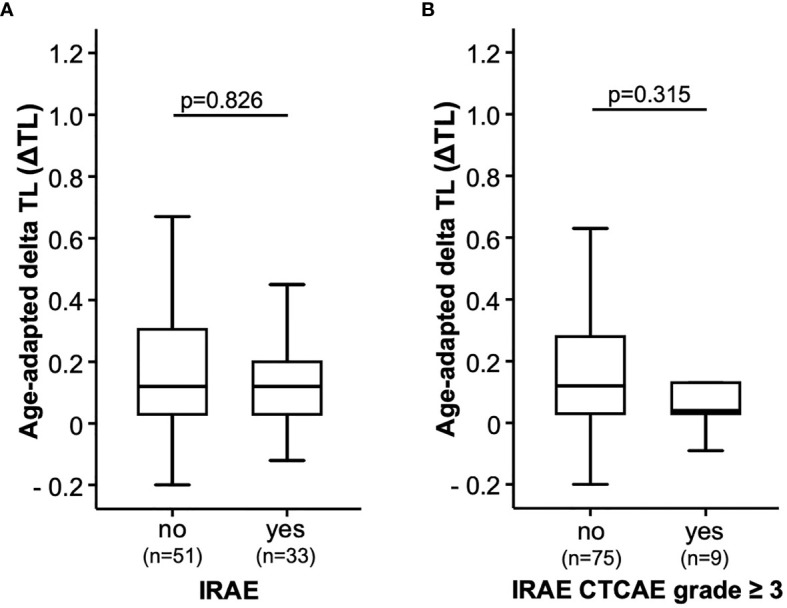
Immune-related adverse events during immune checkpoint inhibitor therapy. The age-adapted delta telomere length (ΔTL) is shown **(A)** in a group of patients without (no) or with (yes) immune-related adverse events (IRAEs). **(B)** In addition, ΔTL is shown in patients with severe IRAEs > grade III (yes) and in those patients who do not (no).

We finally evaluated whether the individual ΔTL was associated with the occurrence of IRAEs during ICI therapy. IRAEs were observed in 33 patients (39.3%). Of these, 9 IRAEs were classified as a severe side effect grade ≥3 CTCAE. However, when we compared the initial ΔTL between patients with or without IRAE/severe IRAE, we did not observe any significant differences (**Figures 4A, B**).

## Discussion

Despite of the broad application of ICIs in various tumor entities, few factors have been established predicting treatment response and OS to ICI treatment (30). TL represents an established biomarker in various hematological diseases (13, 19–21). The impact of TL on the survival of patients under ICI therapy has not been investigated before. In our study, we were able to demonstrate that patients with longer age-adapted ΔTLs had an improved OS compared to patients with shorter ΔTL, independent of the underlying malignant disease. Of note, we observed no differences in treatment response to ICI therapy with respect to patients’ ΔTL. Furthermore, we were not able to demonstrate an association between ΔTL and tumor type, stage of disease, age, gender, previous treatments of patients or IRAEs. We could not observe the previously described shorter ΔTLs of smokers compared to non-smokers possibly due to the small sample size (31). However, we were able to confirm previous data showing that an increased body-mass-index (BMI) had a positive impact on OS in patients receiving ICIs (32).

In the present study using univariate and multivariate analyses, we show that the individual ΔTL in our cohort with heterogeneous solid tumors had a significant impact on the patients’ OS. This effect was most significant when using an ideal ΔTL cut-off value of 0.17. At this cut-off value, the median OS in patients with low ΔTL was only 5.7 months compared to 18.0 months in patients showing a ΔTL above this cut-off value. Since ΔTL is not specifically correlating with known factors for survival as e.g., tumor stage, tumor entity, performance score or response to treatment, we explain the differences in the survival curves with the fact that TLs might represent a possible marker for the assessment of biological age. Due to the relatively small subgroup sizes, no tumor entity-specific analyses were performed. Furthermore, we cannot establish a clear association with ICI therapy because we lack a comparison group receiving any other type of treatment, such as chemotherapy.

Different studies have shown in the past that telomeres shorten as part of the physiological aging process and TL of peripheral blood leukocytes represent an established global marker for the aging process of the entire organism (12, 18). Supporting our hypothesis of shorter TL as marker for the older biological age, previous studies demonstrated that within the general population, shortened TLs are associated with increased all-cause mortality (33). In oncological patients, a comprehensive study including over 47,000 cancer patients revealed that TL correlates with the outcome of cancer patients (34). In line with this observation, performance status scales like “ECOG” or the “Karnofsky-Index” as an indirect marker for biological fitness were shown to predict survival in oncological patients (35). Furthermore, it was previously shown that also immune cell ageing based on different markers was associated with increased mortality (36). We therefore speculate that shorter TLs will also be associated with an increased non-tumor related mortality in our collective as a possible cause for observed difference in OS. This effect could be amplified by pre-existing diseases and tumor-related complications. However, we have insufficient information regarding the specific cause of death of our patients or detailed information about patient’s pre-existing diseases to definitively prove our hypothesis.

An open question in the field is whether additional factors can specifically cause TL shortening in peripheral blood leukocytes. A variety of environmental factors or chronic diseases can in addition accelerate the physiological telomere related aging process in leukocytes (37, 38). In addition, tumor proinflammatory milieu and its microenvironment may also contribute to TL shortening. Data suggest that tumor-associated inflammation can lead to age-independent immune senescence with consecutive shortening of the TL (39, 40). Due to the old age of our cohort, congenital defects in telomerase-associated proteins leading to impaired telomere maintenance seem to be unlikely as major factor.

Lymphocytes are crucial cells for tumor defense and are substantially involved in maintaining an anti-tumoral immune response, mainly within the context of ICI therapy (1). It is tempting to speculate that premature aging in the T-cell compartment results in immune cell exhaustion explaining the impaired OS in patients with shorter TL (41). This issue becomes of particular interest since preliminary work has shown that telomere shortening can predominantly occur in specific immune cell subpopulations, e.g., in memory cytotoxic T cells (42). Due to methodological constraints, we only measured ΔTL of all leukocytes, and it is quite possible that important differences in ΔTL that may predict treatment efficacy are only evident in subpopulations, such as lymphocytes. In the case of immune cell exhaustion, we would first expect a shortening of the ΔTL in the lymphocyte compartment. However, it is therefore all the more surprising that we can already detect differences in survival in a small cohort and with a global screening of ΔTL. Although our study did not demonstrate an inferior response to ICI in patients with shorter TL, the significantly shortened survival time indicates that we may not have captured (small) differences in treatment response or IRAEs due the small patient cohort as well as the selection bias of our patient population. It is possible that subtle differences may be hidden by our basked study design.

Our study has some limitations as the analyzed patient cohort is heterogenous including patients with different tumor entities who receive different ICIs. We also did not include patients who were treated with different therapeutic approaches such as chemotherapy, radiotherapy, or surgery. Moreover, validation of our exploratory findings in larger patient cohorts, that we hope to have stimulated with our study, is essential before a potential clinical implementation can be considered.

## Conclusion

Together, in a collective of 84 patients who underwent therapy with ICIs, we observed that ΔTL at treatment initialization was an independent predictor of OS. We explain our findings with a hypothetical older biological age represented by a premature TL shortening. The pathophysiological background of our observation appears to be unclear. An impact of TL on therapy response or on therapy-associated side effects could not be proven. Additional studies are needed to analyze if further mechanisms that lead to TL shortening in patients with solid tumors exist and to find out if long TLs, especially in lymphocytes, support immunosurveillance competence under ICI therapy during long-term observations.

## Data Availability Statement

The raw data supporting the conclusions of this article will be made available by the authors, without undue reservation.

## Ethics Statement

The studies involving human participants were reviewed and approved by Ethics committee of the University Hospital RWTH Aachen, Germany (approval EK 206/09). The patients/participants provided their written informed consent to participate in this study.

## Author Contributions

SL, TL, BR, and FB designed the study. JG and SL recruited patients. BR and AA performed experiments. SL, JG, FB, and BR performed statistical analysis and generated Figures and Tables. MT, CR, MV, FT, FH, TB, and TL provided intellectual input. BR, SL, JG, FB, and TL drafted the manuscript. SL, BR, FB, and JG revised the manuscript. All authors contributed to the article and approved the submitted version.

## Funding

This publication is part of a project that has received funding from the European Research Council (ERC) under the European Union’s Horizon 2020 research and innovation program (Grant agreement No. 771083). The lab of TL was further supported by the German Cancer Aid (Deutsche Krebshilfe 110043 and a Mildred-Scheel-Professorship), the German-Research-Foundation (SFB-TRR57/P06, LU 1360/3-1, CRC1380/A01, and CA 830/3-1), the Ernst-Jung-Foundation Hamburg, the IZKF (interdisciplinary centre of clinical research) Aachen and a grant from the medical faculty of the RWTH Aachen. Margherita Vieri receives funding from the “Aachener Krebs- und Leukämiehilfe”. FB was funded by a START Grant (No. 691743, RWTH Aachen University) and part of the work was supported by a grant of the “Württembergischer Krebspreis 2019” to FB.

## Conflict of Interest

The authors declare that the research was conducted in the absence of any commercial or financial relationships that could be construed as a potential conflict of interest.

## Publisher’s Note

All claims expressed in this article are solely those of the authors and do not necessarily represent those of their affiliated organizations, or those of the publisher, the editors and the reviewers. Any product that may be evaluated in this article, or claim that may be made by its manufacturer, is not guaranteed or endorsed by the publisher.

## Abbreviations

BMI, body mass index; CR, complete response; CTCAE, common terminology for adverse events; DC, disease control; ECOG PS, eastern cooperative oncology group performance status; GI, gastrointestinal cancers; HNC, head and neck cancers; ICI, immune checkpoint inhibitor; IRAE, immune-related adverse events; MM, malignant melanoma; nDC, no disease control; NSCLC, non-small cell lung cancer; OS, overall survival; PD, progressive disease; PD-1, programmed cell death protein 1; PD-L1, programmed cell death ligand 1; PR, partial response; SD, stable disease; TL, telomere length; ΔTL, (age-adapted) delta telomere length; UC, urothelial carcinoma; UICC, union for international cancer control.
